# A 60 GHz Slotted Array Horn Antenna for Radar Sensing Applications in Future Global Industrial Scenarios

**DOI:** 10.3390/mi15060728

**Published:** 2024-05-30

**Authors:** Binyi Ma, Jing Li, Yu Chen, Yuheng Si, Hongyan Gao, Qiannan Wu, Mengwei Li

**Affiliations:** 1 School of Instrument and Electronics, North University of China, Taiyuan 030051, China; mabinyi99@163.com (B.M.); lijing@tit.edu.cn (J.L.); cy13833876975@163.com (Y.C.); 18834372826@163.com (Y.S.); 2 School of Instrument and Intelligent Future Technology, North University of China, Taiyuan 030051, China; 13781790790@163.com; 3 Academy for Advanced Interdisciplinary Research, North University of China, Taiyuan 030051, China; 4 Center for Microsystem Intergration, North University of China, Taiyuan 030051, China; 5 School of Semiconductors and Physics, North University of China, Taiyuan 030051, China; 6 Key Laboratory of Dynamic Measurement Technology, North University of China, Taiyuan 030051, China

**Keywords:** slot array, horn antenna, millimeter-wave, wide bandwidth coverage, high gain

## Abstract

This paper presents the design of a 60 GHz millimeter-wave (MMW) slot array horn antenna based on the substrate-integrated waveguide (SIW) structure. The novelty of this device resides in the achievement of a broad impedance bandwidth and high gain performance by meticulously engineering the radiation band structure and slot array. The antenna demonstrates an impressive impedance bandwidth of 14.96 GHz (24.93%), accompanied by a remarkable maximum reflection coefficient of −39.47 dB. Furthermore, the antenna boasts a gain of 10.01 dBi, showcasing its outstanding performance as a high-frequency antenna with a wide bandwidth and high gain. To validate its capabilities, we fabricated and experimentally characterized a prototype of the antenna using a probe test structure. The measurement results closely align with the simulation results, affirming the suitability of the designed antenna for radar sensing applications in future global industrial scenarios.

## 1. Introduction

In recent times, there has been a notable surge in demand for radar sensors in the realms of automotive, industrial, and medical applications [1,2,3]. However, conventional radar sensors [4,5] are plagued by challenges including low operational frequencies, elevated costs, restricted bandwidth, and substantial form factors. To address these challenges, the 60 GHz frequency band emerges as a compelling solution due to its inherent broadband characteristics. Moreover, sensors operating in the 60 GHz band have the capability to capture rich and exceptionally accurate point cloud data [6,7], a critical factor for sustaining precision in measurements across a spectrum of applications within the global industrial radar sensing domain. Therefore, antennas characterized by both high gain and wide bandwidth [8,9] in this frequency band are poised as promising alternatives for advancing global industrial radar [10,11] sensing applications in the future. Currently, SIW technology [12,13,14] is gaining maturity within the high-frequency antenna domain [15]. An SIW represents a dielectric-filled metallic waveguide with a planar structure. In contrast to conventional metallic waveguides [16,17], an SIW offers distinct advantages such as a reduced profile, minimal signal loss, and seamless integration with planar circuits. As a result, SIW structures have found extensive utility in both circuit and antenna designs within the MMW frequency bands [18,19].

In 2015, S. Ramesh and his team conducted pivotal research in indoor radio link characterization for millimeter-wave wireless communications, utilizing dielectric-loaded index-tapered slot antennas [20]. This design achieved a 3.33 GHz bandwidth, 6.45 dBi gain, and a maximum reflection coefficient of 21.31 dB. However, with the evolution of high-speed communication technology [21,22], its bandwidth and gain limitations became apparent, highlighting the need for more advanced designs. By 2019 [23], Nanda Kumar Mungaru and Thangavelu Shanmuganantham had addressed these limitations with a broadband H-spaced head-shaped slot antenna based on an SIW, specifically for 60 GHz wireless communication. This innovative antenna showed an impressive impedance bandwidth of 3.22 GHz, a reflection coefficient of −38.3 dB, and a gain of 6.812 dBi. In the same year, another significant advancement was a substrate-integrated waveguide-based slot antenna, also for 60 GHz applications [24]. It boasted a 3.64 GHz impedance bandwidth, a reflection coefficient of −21.31 dB, and a 6.812 dBi gain. Compared to S. Ramesh et al.’s earlier design, these newer antennas offered an improved bandwidth and reflection coefficient [25]. Moving to 2021, T. Shanmuganantham and colleagues analyzed a tree-shaped slotted impedance-matching antenna for 60 GHz femtocell applications. They achieved remarkable improvements in size, gain, and bandwidth, with a 7.46 dBi gain and a 9.7 GHz bandwidth, surpassing S. Ramesh’s earlier design. Despite these advancements, the challenge of integrating these technologies into future radar sensing applications within the global industrial landscape remained formidable. In 2023, Manish Sharma introduced a Conformal ultra-compact narrowband 60.0 GHz four-port millimeter-wave MIMO antenna for wearable short-range 5G application [26]. This state-of-the-art design featured a compact size of 16 × 16 × 0.254 mm^3^, a 1.735 GHz bandwidth, and a maximum reflection coefficient of −35.79 dB, exhibiting superior gain. However, the trade-off for this enhanced gain was a narrower bandwidth, potentially limiting its electrical performance stability near the central frequency [27].

Addressing the critical challenge of low gain and narrow bandwidth in antenna designs for radar industry applications, this paper introduces a novel 60 GHz slot array horn antenna based on an SIW structure. This design markedly enhances overall antenna performance by meticulously crafting the radiation band structure and gap array. The resultant antenna demonstrates exceptional capabilities, notably in terms of gain and bandwidth. The advanced performance of this antenna is pivotal for radar sensors, enabling them to achieve high accuracy and generate rich point cloud data. These attributes make the proposed antenna an optimal choice for future radar sensing applications across various global industrial scenarios. By overcoming the limitations of previous designs, this antenna stands as a significant step forward in the field, offering a robust solution tailored to meet the demanding requirements of modern radar technology.

## 2. Design and Simulation

**Figure 1:**
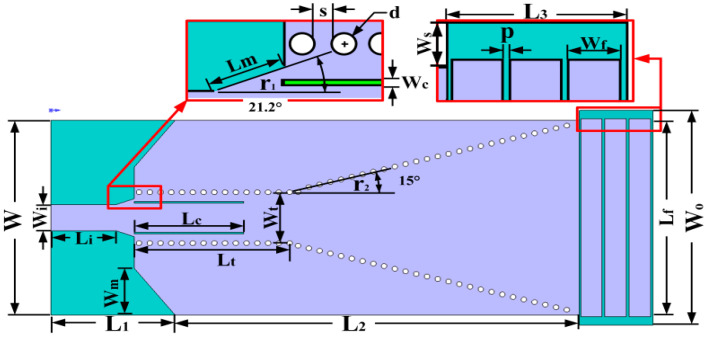
Antenna design parameters.

In this study, we have developed an antenna substrate using Arlon AD255C (Chandler, AZ, USA), a high-frequency dielectric material, featuring copper layers on both the top and bottom surfaces to facilitate electromagnetic radiation. The copper layer of the metal substrate has a thickness (*h_p_*) of 17.5 μm and a conductivity (*δ*) of 5.8 × 10^7^ s/m; its relative permittivity is 2.55, with a dielectric loss tangent of 0.0014, optimizing its performance for high-frequency applications. Additionally, our study employed Ansys Electronics Desktop 2022 R1 software for structural simulation. Comprehensive simulations and optimizations were conducted to refine the antenna’s performance. The result is an SIW slot array [28,29] horn antenna, which notably achieves a bandwidth of 14.96 GHz, a gain of 10.01 dBi, and is tuned to a resonant frequency of 60 GHz. The detailed design and specifications of this antenna are illustrated in Figure 1.

Our study aims to iteratively refine and optimize the design of the antenna described above. Next, we will begin describing the following sections:

### 2.1. SIW Structure Design

The antenna designed in this paper adopts the traditional rectangular waveguide [30,31] and the primary mode in the rectangular waveguide TE_10_ mode [32]. It is also the lowest mode under the mode of TE_mn_; the wavelength of $\lambda_{g}$ is an important parameter of the rectangular waveguide mode of TE_10_ and can be expressed as below:

(1) $$\lambda_{g}=\frac{\lambda }{\sqrt{\varepsilon_{r}-{\left(\lambda /\lambda_{c}\right)}^{2}}}$$

The column width of the metal through the hole of *W_t_* can be obtained by using the traditional rectangular waveguide calculation formula. The width of the SIW is obtained by the following Formula (2):

(2) $$W_{t}=W_{i}+\frac{d^{2}}{0.95S}$$

The diameter (*d*) to a width of waveguide (*W_t_*) is not mentioned in Formula (3) and sometimes it will give error values, and the more appropriate equation is represented as:

(3) $$W_{i}=W_{t}+1.08\times \frac{d^{2}}{S}-0.1\times \frac{d^{2}}{W_{i}}$$

Here, *W_i_* represents the equivalent width of the SIW. Upon obtaining the equivalent width *W_i_* of the SIW, we can determine the cutoff frequency of the SIW in the TE_10_ mode, as shown in Formula (4):

(4) $$f_{\mathrm{TE}10}=\frac{1}{2W_{i}\sqrt{\mu \varepsilon }}=\frac{c}{2W_{i}\sqrt{\mu_{r}\varepsilon_{r}}}$$

Here, c represents the speed of light in a vacuum, and $\mu_{r}$ and $\varepsilon_{r}$ denote the relative permeability and relative permittivity of the substrate material.

Based on the above information, the dimension design of the SIW structure can be completed. First, it is necessary to choose an appropriate width *W_i_* for the SIW based on the operating frequency. Taking into account the radiation leakage problem and the difficulty of machining, the size and spacing of the metallized through-holes [25] should satisfy conditions $d\leq \frac{\lambda_{g}}{5}$, S≤2d, and $d<0.1\times W_{t}$; S and d were chosen to be 0.6 mm and 0.3 mm, respectively. The microstrip [33] part is combined with the conical corner of λ4 proposed in Ref. [24] to better radiate electromagnetic waves into the SIW rectangular waveguide, where the inclination (r_1_) of the conical corner of the antenna transmission line is 21.2°. The horn aperture (r_2_) is selected to be 30° to better radiate electromagnetic waves outward.

**Figure 2:**
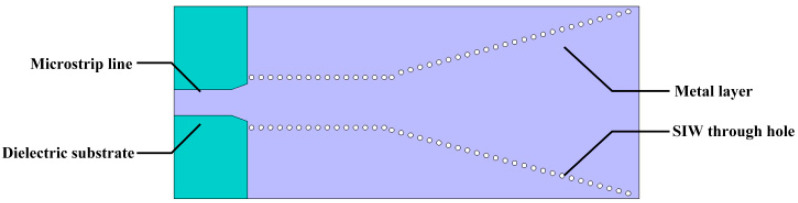
SIW horn antenna structure.

**Figure 3:**
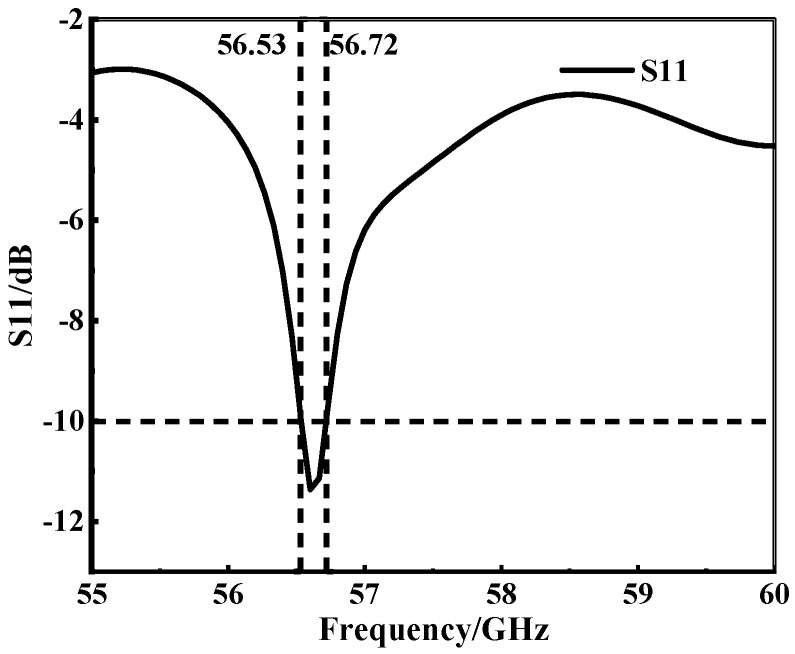
SIW horn antenna bandwidth performance.

The antenna structure is shown in Figure 2. The SIW column width of the antenna is 3.383 mm, and the substrate thickness is 0.508 mm. Through simulation, the bandwidth performance of the antenna is shown in Figure 3, and the antenna’s bandwidth as designed in step 1 is 0.19 GHz.

### 2.2. Antenna Radiation Belt Design

**Figure 4:**
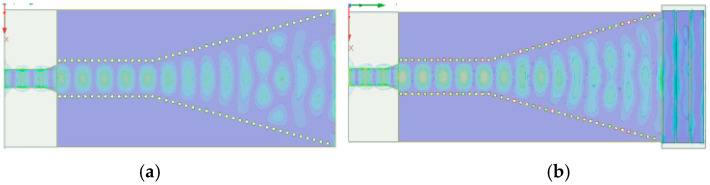
(**a**) Electromagnetic radiation without radiation belt; (**b**) electromagnetic radiation with radiation belt.

The initial iteration of the SIW horn antenna, as developed through the aforementioned steps, exhibited a bandwidth of only 0.19 GHz. This was significantly narrower than the design requirements, with the center frequency and other parameters not aligning with our goals. To address this issue, we conducted an in-depth analysis of the antenna’s electromagnetic radiation characteristics [34,35]. We discovered that the return loss at the transmitting end of a standard SIW horn antenna was notably high, negatively impacting its electromagnetic radiation, as depicted in Figure 4a. To optimize the electromagnetic performance of the antenna, we introduced a novel design element: the radiation belt, as illustrated in Figure 4b.

**Figure 5:**
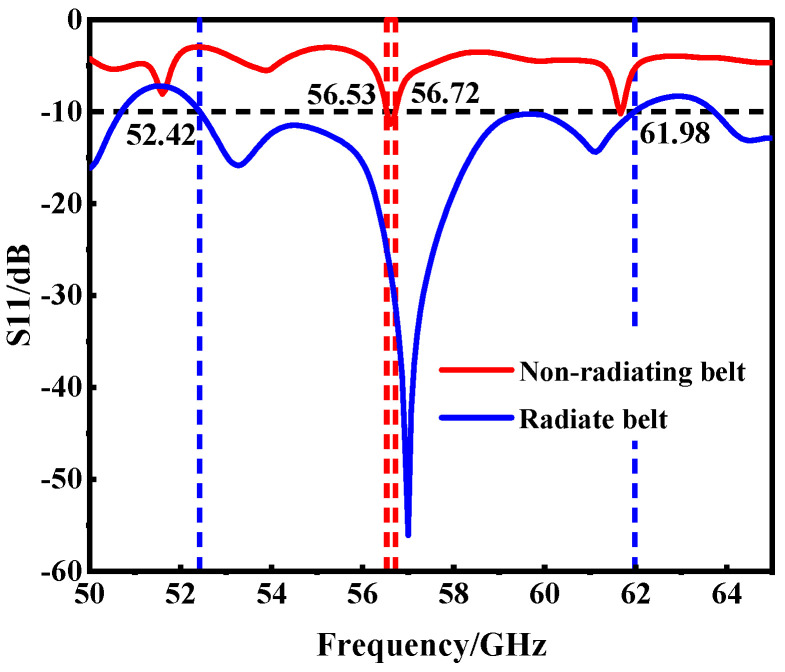
Results with or without radiation belt S_11_.

This strategic modification was found to not only optimize the electromagnetic radiation of the antenna but also substantially enhance its bandwidth performance. The improvements achieved through this design adjustment are clearly demonstrated in the parametric metrics shown in Figure 5. Most notably, the introduction of the radiation strip significantly expanded the antenna’s bandwidth from a mere 0.19 GHz to an impressive 9.56 GHz. This dramatic increase marks a considerable advancement in the bandwidth performance of the antenna, aligning it more closely with the stringent requirements of high-frequency applications.

### 2.3. Antenna Slot Array

Under the TE_10_ mode of a rectangular waveguide, opening narrow slots along the direction of current flow generally does not significantly affect the original mode. However, when etching slots on the metal surface, if the slots disrupt the original surface conduction current, displacement currents (i.e., time-varying tangential electric fields) will be induced on the aperture formed by the slots to maintain current continuity. In this case, the open slots are excited, leading to the radiation of electromagnetic energy into space.

According to the principle of electromagnetic equivalence, the radiation of an ideal slot with a length of $L_{c}$ and a width of Wc(Lc=λ2,Wc≤Lc) is equivalent to a magnetic current source $J_{m}$. Following Babinet’s principle, it complements a strip dipole with the same shape. Because the rectangular substrate-integrated waveguide (SIW) has transmission characteristics similar to those of traditional rectangular metal waveguides filled with dielectrics, the design methods for SIW slot antennas and traditional metal waveguide slot antennas are essentially the same. However, since the SIW is fabricated using PCB technology, its thickness is relatively thin, and the narrow sidewalls of the traditional metal waveguide are equivalent to the through-hole metallization of the SIW. Therefore, radiation can only be achieved by opening slots on the upper and lower metal surfaces of the SIW.

**Figure 6:**
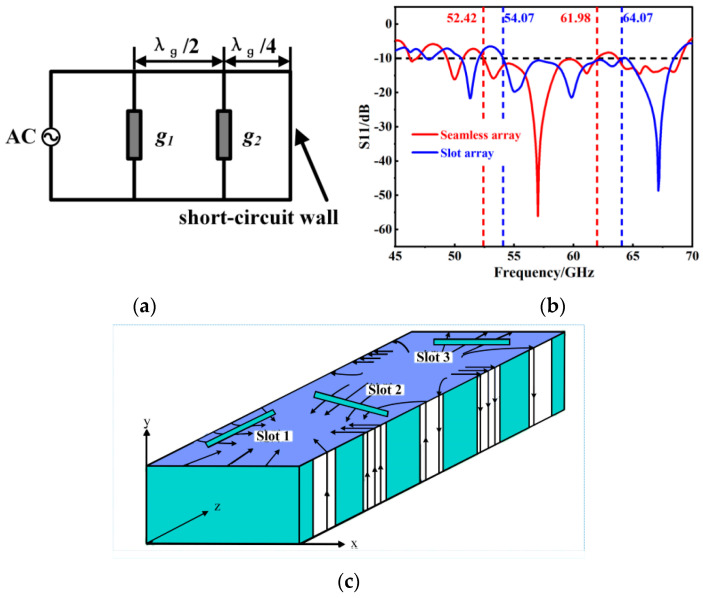
(**a**) Three slot types of the SIW TE_10_ mold; (**b**) results with or without slot and S_11_; (**c**) three types of slots under TE_10_ mode in SIW.

For the SIW operating in the TE_10_ mode, it exhibits three common types of slots based on their positions, as illustrated in Figure 6c. Slot 1 represents a longitudinal slot on the wide side, parallel to the propagation direction of the SIW. To cut off the current distribution, it needs to be offset from the centerline of the SIW by a certain distance. Slot 2 is a diagonal slot on the wide side, with its center located on the symmetrical centerline of the SIW and at an angle with respect to the propagation direction. Slot 3 is a transverse slot on the wide side, perpendicular to the propagation direction. Among these, the longitudinal slot array on the wide side is the most common type of SIW slot array. When the current is interrupted to open slots, energy leaks into space, thereby radiating electromagnetic waves. Therefore, by establishing a slot array, electromagnetic wave radiation can be controlled to achieve an increase in bandwidth.

In the quest to further augment the performance of the antenna, the concept of a slot array mode, as indicated in Reference [36], was incorporated in step 3 of our process. The SIW, fabricated using the PCB process [37], possesses a thin structure. This thinness is analogous to the narrow sidewalls of a traditional metal waveguide, achieved through metalized vias or holes. In the PCB design, electromagnetic radiation is primarily confined to the top and bottom wide edges of the SIW, where the metal layers are present. A critical aspect of this design is the presence of gaps within the SIW structure. These gaps disrupt the continuity of the transverse surface currents, leading to a distinct behavior. Specifically, the transverse surface currents are redirected towards the ends of these gaps. This redirection results in abrupt changes in the longitudinal currents along the propagation direction of the SIW, as detailed in References [38,39]. Consequently, the longitudinal slot on the wide side of the SIW can be effectively modeled as an admittance in parallel with the transmission line. This concept is instrumental in establishing the equivalent circuit model, depicted in Figure 6a.

When the wide-side longitudinal slot of the SIW is in resonance, Stevenson provided its normalized conductance as:

(5) $$g=2.09\frac{W_{i}}{h}\frac{\lambda_{g}}{\lambda }cos^{2}\left(\frac{\pi \lambda }{2\lambda_{g}}\right)sin^{2}\left(\frac{\pi \lambda }{W_{i}}\right)$$

Figure 6b illustrates the significant impact of implementing a slot array on the bandwidth of the antenna. This comparison clearly shows that introducing the slot array not only extends the bandwidth from 9.56 GHz to a remarkable 10 GHz, but also beneficially shifts the center frequency towards the higher frequency spectrum, moving from 57.2 GHz to 59.07 GHz. This shift brings the antenna’s performance closer to the targeted center frequency of 60 GHz, aligning more closely with our design objectives.

**Figure 7:**
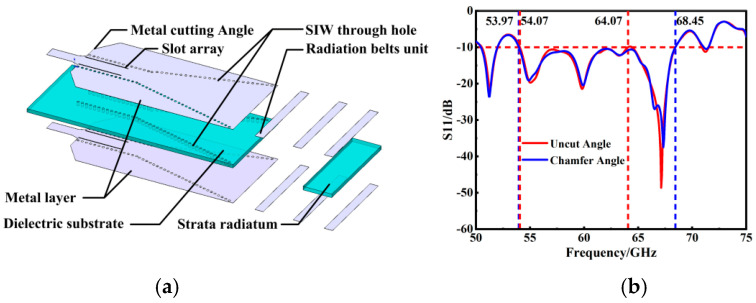
(**a**) Three-dimensional explosion diagram of the overall antenna structure; (**b**) results of cutting angle S_11_ with or without metal layer.

In addition to the slot array modification, another critical strategy for optimizing the antenna’s electromagnetic performance involves reducing the area of the metal layer. This is achieved by cutting the metal at specific angles, as discussed in Reference [40]. Reducing the metal layer area minimizes metal radiation and coupling effects, thereby enhancing the electromagnetic properties of the antenna. The simulations and comparisons of the S_11_ results provide insightful evidence of this improvement. Notably, the bandwidth’s left and right boundaries at the metal layer’s tangent angle are expanded outwards, thereby broadening the overall bandwidth from the initial 10 GHz to an impressive 14.48 GHz. The culmination of these design modifications and optimizations is the finalized SIW slot array horn antenna, as showcased in Figure 7a. This design represents the integration of innovative techniques to significantly improve the antenna’s bandwidth and electromagnetic performance, marking a substantial advancement in antenna technology for high-frequency applications.

## 3. Analysis, Results, and Discussions

After the above three stages of the design process, the overall structure of the antenna is finally obtained. The effects of the geometric parameters are investigated in this part. The following parameters are selected for this study: the length (*L_c_*) and width (*W_c_*) of the slot array, the distance (*P*), length (*L_f_*), and width (*W_f_*) of the radiation belt unit, and the width (*W_m_*) of the metal cutting angle. However, the parameters described above will exhibit a hierarchical relationship. The initial design parameters of the antenna are as follows (Table 1).

**Figure 8:**
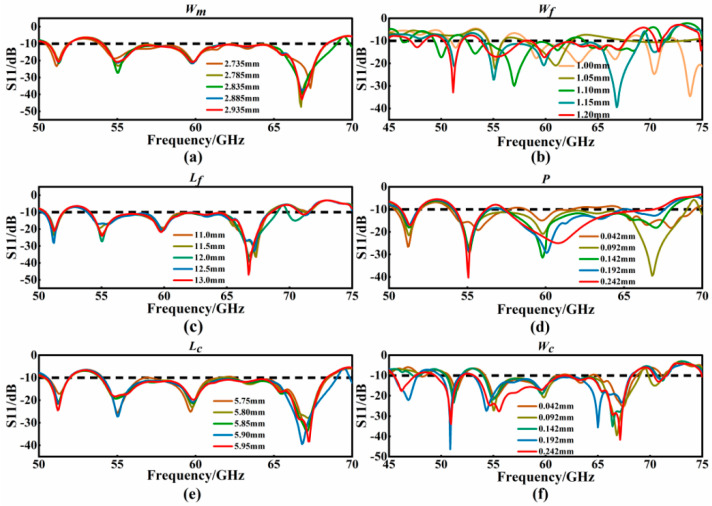
Antenna parameter optimization diagram. (**a**) the impact of the metal cutting angle width (*W_m_*) on antenna performance. (**b**) the impact of the radiation belt unit width (*W_f_*) on antenna performance. (**c**) the impact of the radiation belt unit length (*L_f_*) on antenna performance. (**d**) the impact of the radiation belt unit distance (*P*) on antenna performance. (**e**) the impact of the slot array length (*L_c_*) on antenna performance. (**f**) the impact of the slot array width (*W_c_*) on antenna performance.

The scan optimization and discussion of the above parameters are as follows (Figure 8).

This part of the study focuses on the value of S_11_ under different *W_m_* values. A step scanning optimization method was employed, incrementing *W_m_* by 0.02 mm steps. The simulation results revealed that as *W_m_* increased, the optimum value was identified at 2.835 mm. At this width, the antenna achieved its widest bandwidth, spanning 14.96 GHz (from 54.06 GHz to 69.02 GHz), with the maximum reflection coefficient reaching −39.27 dB. After careful analysis, a *W_m_* of 2.835 mm was determined to be the most effective for achieving optimal bandwidth and reflection characteristics.

The study then analyzed the impact of varying the *W_f_* on the antenna’s performance, specifically looking at the S_11_ parameters. It was observed that the antenna’s bandwidth shifted at both ends as *W_f_* increased. The most favorable bandwidth expansion occurred at a *W_f_* of 1.15 mm. Meanwhile, when *W_f_* is set to 1.15 mm, the resonance frequency aligns well with the *W_m_* at 2.835 mm, yielding optimal bandwidth. Consequently, an optimal antenna performance was achieved when the width of the *W_f_* was set to 1.15 mm.

This part of the study examines the S_11_ results for different *L_f_* values. The simulations indicate that the S_11_ reflection coefficient peaks at an *L_f_* of 13 mm. However, the optimal length for *L_f_* is determined to be 12 mm. At *L_f_* = 12 mm, the length of the radiation belt closely aligns with the aperture of the SIW horn antenna, facilitating more effective radiation of electromagnetic waves. To minimize electromagnetic wave scattering, the width of the radiation belt is designed to be larger than the distance between the two metallized holes in the SIW horn antenna layer. The study further investigates how the variation in the *P* affects the antenna’s reflection coefficients. It was observed that the antenna’s bandwidth decreases as *P* increases, particularly when the band spacing exceeds 0.15 mm. The antenna achieves its optimal impedance bandwidth of 14.96 GHz at *P* = 0.15 mm, marking this as the best value for antenna bandwidth performance.

This segment of the study focuses on the influence of the *L_c_* on the antenna’s bandwidth. It was observed that the antenna’s bandwidth reaches its maximum of 14.96 GHz when *L_c_* is set to 5.90 mm. Consequently, an *L_c_* of 5.90 mm was identified as the optimal length for maximizing the antenna bandwidth. The study then examines how varying the *W_c_* affects the antenna’s impedance bandwidth. The results show a gradual widening of the antenna’s impedance bandwidth with an increase in *W_c_*, with the maximum bandwidth of 15.15 GHz achieved at a *W_c_* of 0.192 mm. Therefore, the optimal width for the slot array is determined to be 0.192 mm, as it yields the highest bandwidth performance.

**Figure 9:**
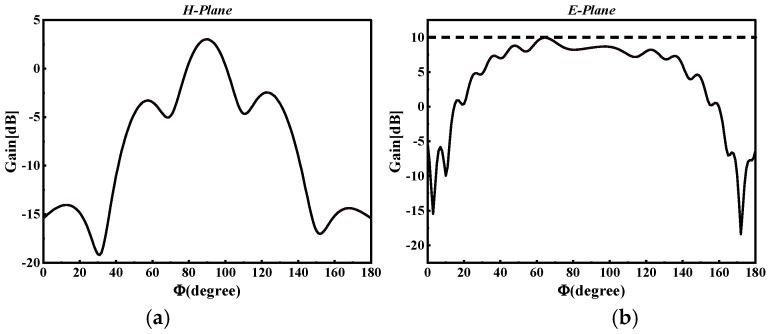
Radiation patterns of E and H planes of SIW slot array horn antenna; (**a**) H-plane radiation pattern; (**b**) E-plane radiation pattern.

**Figure 10:**
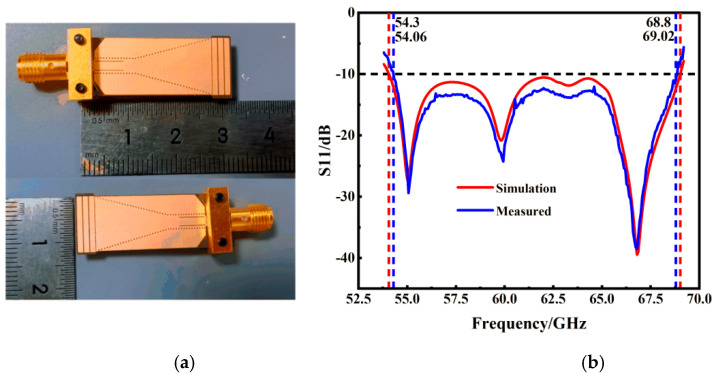
Antenna diagram and performance verification; (**a**) picture of real products; (**b**) analog and measurement bandwidth.

Following the meticulous optimization of the core parameters of the antenna, we achieved notable results as depicted in Figure 9, which displays the gain exponent. The extensive simulation process culminated in the antenna achieving a significant bandwidth of 14.96 GHz along with a substantial gain of 10.01 dBi. These figures mark a considerable advancement in antenna performance, particularly in terms of bandwidth and gain. The detailed bandwidth simulation, showcasing these results, is presented in Figure 10b. This diagram illustrates the effective bandwidth coverage of the antenna, confirming its suitability for high-frequency applications. The substantial bandwidth, coupled with the high gain, indicates that the antenna is well-equipped to meet the demanding requirements of contemporary wireless communication systems. The specific size parameters of the designed 60 GHz SIW slot array horn antenna are given in Table 2:

The final design of the SIW slot array horn antenna, as depicted in Figure 10a, was meticulously crafted and manufactured based on the optimized parameters previously outlined. The antenna boasts a compact size of 32 × 13 × 0.543 mm^3^, highlighting its small footprint and potential for seamless integration into various applications. The comprehensive simulation and validation results of this antenna are presented in Figure 10b. These results confirm its performance metrics and suitability for the intended high-frequency applications. The combination of its small size and the results from the simulation and validation processes underscore the antenna’s innovative design and its potential impact in the field of wireless communication.

The measurement results of the SIW slot array horn antenna revealed a bandwidth of 14.5 GHz and a reflection coefficient of −37.51 dB. These figures are largely in alignment with the theoretical predictions, affirming the success of the antenna design. However, a minor shift in frequency was observed, which warrants consideration.

This deviation from the theoretical values is likely attributable to a few key factors encountered during the manufacturing and testing phases. Firstly, errors in the positioning and sizing of the through-hole apertures during the manufacturing process could have contributed to these discrepancies. Secondly, the loss of joints in the measurement process is another potential source of variation. Lastly, environmental conditions [41] during testing may have had an impact on the results. These factors underscore the complexities involved in translating theoretical designs into practical applications. They highlight the need for precision in manufacturing and the influence of external conditions on the performance of high-frequency antennas.

## 4. Comparison

Table 3 presents a comprehensive comparison of the antenna’s performance parameters across the three distinct design phases discussed in Section 2. This comparative analysis clearly illustrates that the antenna exhibits superior performance in the third phase compared to the first two phases. The enhanced performance in the final phase can be attributed to the strategic additions made to the antenna design, namely the incorporation of radiation bands and slot arrays. These modifications have proven to be effective in significantly improving both the bandwidth and gain of the antenna. This improvement highlights the importance of iterative design and optimization in antenna engineering, demonstrating how each phase of development contributes to the overall enhancement of antenna performance.

Table 4 effectively compares the performance of the antenna developed in this paper with other antennas previously reported in the literature. The comparison is based on key parameters such as bandwidth, gain, size, and reflection coefficient. The findings indicate that the antenna designed in this study holds a distinct advantage in terms of bandwidth performance, surpassing most of the other antennas. Additionally, it demonstrates superior performance in terms of reflection coefficient, and its gain and size are also comparatively better than most of the antennas in the study.

The excellence in these performance metrics is particularly significant for industrial radar sensing systems. The wide bandwidth of the antenna contributes to a higher distance resolution, which is crucial in accurately determining the position of objects. Moreover, the center frequency of 60 GHz enhances the velocity resolution, enabling more stable detection of moving objects. The combination of a wide bandwidth and an optimal center frequency is vital for collecting rich and highly accurate point cloud data. This, in turn, facilitates the generation of high-resolution radar images, crucial for the efficacy of industrial radar systems.

## 5. Conclusions

This study focused on the development of a 60 GHz slot array horn antenna, leveraging an SIW structure. A key aspect of this design was the strategic control of electromagnetic radiation direction through the arrangement of apertures. Additionally, the antenna’s bandwidth performance was optimized by incorporating a radiation band at the radiation front. The use of a slot array further expanded the working bandwidth of the antenna.

Experimental evaluations of the antenna demonstrated its exceptional performance, with a strong correlation between simulation predictions and actual measurement results. The antenna’s impressive performance characteristics are pivotal for providing high-resolution radar imaging, crucial for accurate target detection and tracking in industrial applications. Moreover, the antenna’s compact size contributes to cost reductions in the deployment of radar sensing systems in industrial settings.

In conclusion, the designed antenna, with its combination of excellent performance, high resolution, and cost-effectiveness, emerges as a promising alternative for future industrial radar sensing applications. Its innovative design and successful implementation mark a significant advancement in the field of radar technology.

## Figures and Tables

**Table 1:** Initial values of optimization parameters.

Symbol	Value (mm)	Description
*W_m_*	2.735	Width of metal cut corners
*P*	0.150	Radiation belt unit spacing
*W_f_*	1.000	Radiation band unit width
*L_f_*	11.000	Radiation belt unit length
*W_c_*	0.092	Width of slot array element
*L_c_*	5.750	Length of slot array element

**Table 2:** Specific values of antenna design parameters.

Symbol	Value (mm)	Description
*W*	11.800	Antenna width
*L*_1_	6.705	Transmission line and metal cutting length
*L*_2_	21.795	Horn length
*L*_3_	3.980	Radiation layer length
*W_i_*	1.575	50 Ω feeder width
*L_i_*	3.500	50 Ω feeder length
*W_t_*	3.383	Metallized through hole row wide
*L_t_*	8.405	Rectangular waveguide length
*W_m_*	2.835	Width of metal cut corners
*L_m_*	1.064	Conical corner length
*W_f_*	1.150	Radiation band unit width
*L_f_*	12.000	Radiation belt unit length
*d*	0.300	Metal through hole diameter
*S*	0.600	Metallized through hole aperture
*P*	0.150	Radiation belt unit spacing
*W_c_*	0.192	Width of slot array element
*L_c_*	5.900	Length of slot array element
*W_o_*	13.000	Width of horn antenna output

**Table 3:** Compares the performance of the three phases.

	Bandwidth (GHz)	Gain (dBi)	Maximum Reflection Coefficient (dB)
Phase 1	0.19	5.43	−11.35
Phase 2	9.56	10.56	−56.08
Phase 3	14.96	10.01	−39.47

**Table 4:** Comparison between the published SIW structural antennas at 60 GHz and our work.

Reference	Size (mm)	Gain (dBi)	Bandwidth (GHz)	Return Loss (dB)
[20]	29.5 × 8 × 0.787	10	3.33	−12.23
[23]	14 × 8 × 0.381	/	4.18	−33.95
[24]	14 × 8.4 × 0.381	/	3.5	−21.98
[25]	7 × 12 × 0.508	6.54	6.8	−36.35
[26]	16 × 16 × 0.254	10.56	1.73	<−50.0
[42] (simulation)	17.5 × 14.5 × 0.42	11.8	10	/
[43]	13 × 9 × 0.381	/	5.3	−29.6
This work	32 × 13 × 0.543	10.01	14.5	−37.51

## Data Availability

The data that support the findings of this study are available from the corresponding author upon reasonable request.
